# Digital transformation of care for keratoconus patients: ML modeling structural outcomes of corneal collagen cross-linking

**DOI:** 10.3389/fmed.2025.1462653

**Published:** 2025-06-04

**Authors:** Yauhen Statsenko, Darya Smetanina, Roman Voitetskii, Gillian Lylian Simiyu, Mikalai Pazniak, Elena Likhorad, Aleh Pazniak, Pavel Beliakouski, Dmitri Abelski, Fatima Ismail, Klaus Neidl-Van Gorkom, Milos Ljubisavljevic

**Affiliations:** ^1^Medical Imaging Platform, ASPIRE Precision Medicine Research Institute Abu Dhabi, Al Ain, United Arab Emirates; ^2^Department of Radiology, College of Medicine and Health Sciences, United Arab Emirates University, Al Ain, United Arab Emirates; ^3^Big Data Analytics Center, United Arab Emirates University, Al Ain, United Arab Emirates; ^4^Microsurgery Department, Eye Microsurgery Center “Voka”, Minsk, Belarus; ^5^Department of Pediatrics, College of Medicine and Health Sciences, United Arab Emirates University, Al Ain, United Arab Emirates; ^6^Department of Physiology, College of Medicine and Health Sciences, United Arab Emirates University, Al Ain, United Arab Emirates; ^7^Neuroscience Platform, ASPIRE Precision Medicine Research Institute Abu Dhabi, Al Ain, United Arab Emirates

**Keywords:** keratoconus, corneal collagen cross-linking, CXL outcomes, machine learning models, predictive models, keratometry readings, corneal thickness, precision medicine

## Abstract

**Background:**

Structural outcomes of corneal collagen cross-linking (CXL) have not been thoroughly investigated. Clinical risk assessment would benefit from a reliable prognosis of postoperative minimal (MCT) and central corneal thickness (CCT).

**Objective:**

The objective of this study was to find a combination of diagnostic modalities and measurements that reliably reflect CXL efficiency in terms of corneal thickness.

**Methods:**

We retrospectively reviewed the medical histories of 107 patients (131 eyes) who underwent CXL. The dataset included preoperative examinations and follow-up results, which totalled 796 observations.

**Results:**

The postoperative changes in MCT are more pronounced, clinically relevant, and meaningful than in CCT. MCT should serve as the major clinical marker of corneal thinning after CXL. The cornea's potential to recover reduces in advanced keratoconus. A polynomial curve demonstrates the natural course of corneal remodeling. It includes thinning immediately after CXL and stabilization with partial recovery of corneal thickness over time. Baseline pachymetry data can adequately reflect the outcomes. Preoperative BAD and topographic indices *strongly* correlate with the outcomes. Keratometry and refractometry data exhibit *moderate* associations with postoperative corneal thickness. The models trained on a combination of top correlating features, clinical data, and time after intervention provide the most reliable prognosis.

**Conclusion:**

Risk assessment is accurate with multimodal preoperative diagnostics. A stratification system should take into account findings in different diagnostic modalities.

## 1 Introduction

Keratoconus (KC) is an ectatic corneal disorder leading to visual impairment. KC usually presents in the second or third decade of life with a global prevalence of 138 per 100,000 people (1, 2). The etiology remains unclear: A combination of genetic, biomechanical, and environmental factors may account for disease occurrence (3). KC risk factors include frequent eye rubbing, allergic reactions, and permanent ultraviolet radiation exposure (4). In early stages, the pathology is asymptomatic (5). Corneal topography is a screening technique aimed at promoting early treatment before irregular astigmatism, myopia, and corneal scarring develop (6–8).

Currently, corneal collagen cross-linking (CXL) is the most effective method to halt KC progression by rejuvenating collagen fibril molecules. As a result, KC remains firm for up to 28 years. Although CXL may damage endothelial cells and injure nerves (9), the procedure prevents the severe KC stages that would require corneal transplantation (keratoplasty) (10). Despite a high graft survival rate (90.4%), the method has limitations and side effects (11, 12). Treatment response varies among patients; therefore, risk assessment will enable the delivery of individualized medical care.

Herein, we critically appraise clinical evaluation, pachymetry, visiometry, refractometry, and topography tests for assessing KC progression after the treatment. An ophthalmic examination includes a comprehensive series of tests that indicate the optimal timing of CXL (13). Since KC causes corneal thinning and protrusion, pachymetry readings may help to forecast CXL effectiveness (14). According to recent studies, corneal thickness decreases rapidly within 3 months after CXL and restores to the baseline level within a year (15). Little extension between the outer and inner surfaces of the cornea leads to favorable outcomes (16).

Maximum curvature value (Kmax) is a strong predictor of disease progression and effectiveness of CXL. The smaller the preoperative Kmax is, the more successful the intervention will be (17, 18). The results in the best-corrected visual acuity (BCVA) test also reflect the efficacy of CXL. Recent studies have tried to identify the most accurate predictors of disease progression and patients' responses to interventions. However, they considered a limited number of parameters. To overcome this limitation, we initiated the current project.

## 2 Objectives

*The aim of the current study* is to find and set up a combination of diagnostic modalities and measurements that reliably reflect CXL efficiency. Hypothetically, risk assessment is more accurate with multimodal preoperative diagnostics than unimodal. If this is the case, a risk stratification system should take into account different diagnostic modalities to predict the efficiency of treatment. Alternatively, the study should highlight a diagnostic modality most strongly correlated with treatment outcomes. Knowledge of this modality will allow us to create the desired unimodal risk assessment tool.

The following study adopts the current trend of introducing machine learning (ML) into clinical practice. The predictive ML models foster further development of precision medicine by identifying optimal therapy and individualizing treatment options. In addition to creating models, we want to help ophthalmologists interpret various instrumental findings together with clinicodemographic data. For this, our study will explore the value of keratometry, pachymetry, visiometry, refractometry, and topography tests. These data will help provide a more explicit informative value of the tests, which, in turn, will help improve clinician decision-making.

To achieve the study aim, we formulated and fulfilled the following tasks:

1. Assess the relationships between pre- and postoperative pachymetry findings.
2. Examine the association of CXL outcomes with the results in keratometry, visiometry, refractometry, topography tests, and clinical examination.
3. Model the dynamics in the central corneal thickness (CCT) and minimal corneal thickness (MCT) after CXL for KC.
4. Identify top-informative features of CXL effectiveness in KC care.

## 3 Materials and methods

### 3.1 Study cohort

The KC prevalence largely varies among different populations; therefore, estimating the minimal sample size was challenging. To decide on the number of patients required for the current research, we analyzed recently published reports on CXL outcomes. The number of cases in the original studies depends on the study design, objectives, and resources. Often, pilot studies or preliminary reports assess the safety or explore the initial efficacy of CXL in a small group of patients, from 10 to 30 cases (19–26). Single-center clinical studies typically evaluate outcomes such as corneal stabilization, visual acuity improvement, or changes in keratometry values in a specific patient population of 2–100 (27–30). To provide more robust evidence on the safety, efficacy, and long-term outcomes, multicenter studies and comparative clinical trials include diverse patient populations and may compare different CXL techniques in a large cohort of patients: from 100 to 500 cases (31–33).

We retrospectively reviewed the medical histories of 107 KC patients (131 eyes) who underwent CXL in the medical center “Voka” from January 2018 to December 2022. The study included male and female patients of all ages with a history of KC treated with CXL. Exclusion criteria were age under 16 years, corneal thickness below 400 microns, severe dry eye, other corneal diseases or infections, prior re-CXL, pregnancy, and missing follow-up examinations. We also excluded cases with advanced KC stages, cicatricial corneal changes observed during biomicroscopy, and autoimmune diseases in decompensation. Physicians examined each patient one time before and several times after the intervention. The examination included slit-lamp biomicroscopy, pachymetry, keratometry, and computerized corneal topography tests.

The final dataset contained the results of preoperative examinations and follow-ups, which totalled 796 observations. Males outnumbered females: 79 vs. 28 (73.8% vs. 26.2%). The average age was roughly equal in both sexes: 29 ± 9 vs 29 ± 7 years, respectively. The studied cases had different severity. Our study included patients with subclinical KC (1 case, 0.76%) and those with KC stages 1 through 3-4. Stage 3 KC was observed in approximately one-third of the patients (41 cases, 31.2%), while nearly half had stage 2 or 2-3 KC (30 cases, 22.9%, and 27 cases, 20.61%, respectively). The most severe cases in our cohort were classified as stage 3–4 KC (13 cases, 9.92%).

#### 3.1.1 Diagnostics of keratoconus

In this study, physicians adopted the following primary diagnostic criteria for keratoconus: an elevated corneal curvature with a Kmax value exceeding 47.0 D, asymmetric astigmatism (a difference of more than 3.0 D in the curvatures of the anterior corneal surface between the two principal meridians), corneal thinning at the site of the cone-shaped protrusion less than 490 μm, and BCVA worse than 20/20 (1.0).

To diagnose KC, our team identified where the typically dome-shaped cornea protrudes outward, forming a cone. During corneal topography tests, we searched for specific topographic patterns indicative of KC. These patterns include localized steepening of the cornea in the mid-peripheral region below the corneal midline (34), an asymmetric bow tie pattern with a skewed radial axis (35), a pear-shaped distortion of the central keratoscopy rings with initial steepening in the temporal quadrant (36), and irregular astigmatism with uneven keratoscopy mires.

Recent research has identified keratometry indices as the most reliable parameters for distinguishing between healthy eyes and KC cases (37). Key features of KC include anterior surface elevation readings greater than 12 μm and posterior surface elevation readings exceeding 20 μm in the central cornea.

For borderline cases, the following findings raised suspicion of KC: anterior surface elevation between 6 and 12 μm, posterior surface elevation ranging from 8 to 20 μm, and a KC index (KI) greater than 1.07 (38, 39). A central keratoconus index (CKI) greater than 1.03 served as a diagnostic criterion for definite KC, though not for its subclinical form (40, 41). Pachymetry tests were also used to support the KC diagnosis. An average pachymetric progression index exceeding 1.6 indicated definite KC (39), while a minimal pachymetry value below 450 μm signaled corneal thinning associated with the disease.

Although not part of our study, physicians can evaluate posterior elevation as a reliable diagnostic marker for KC. A maximal elevation difference greater than 12 μm suggests subclinical pathology, while a difference exceeding 16 μm is diagnostic for KC (42). The optimal method for measuring posterior elevation remains debated. The standard approach analyzes the maximum value above the best-fit sphere within the central 5 mm of the posterior cornea (43). For greater accuracy, some researchers recommend measuring posterior elevation at the thinnest point of the cornea (44, 45). While posterior elevation is a valuable diagnostic tool, physicians should not use it in isolation to identify subclinical or clinical KC cases.

#### 3.1.2 Stages of keratoconus

To assess the severity of KC, we followed the classification system originally proposed by M. Amsler in 1938 and later revised by Krumeich in 1998 (46, 47). However, certain cases proved challenging to categorize as different diagnostic parameters often correspond to different stages of the disease. In these instances, we utilized the ABCD grading system, which addresses the shortcomings of the Amsler-Krumeich classification. Specifically, the older system does not incorporate posterior elevation data or visual acuity, relies on apical corneal thickness rather than the thinnest corneal point, and struggles to distinguish between normal and abnormal cases effectively (48, 49). By employing the ABCD system alongside slit-lamp biomicroscopy findings, we overcame these limitations and evaluated cases more accurately (see Table 1).

**Table 1:** Criteria for grading KC in this study.

**Parameters**	**Unit**	**Stage**			
		**1**	**2**	**3**	**4**
**Amsler-Krumeich classification**					
Myopia/Astigmatism	D	<5	5–8	8–10	Not measurable
Maximal corneal curvature, Kmax	D	<48.0	<53.0	<55.0	>55.0
Posterior corneal curvature	D	<59.25	<65.5	<68.5	>68.5
Minimal apical corneal thickness	μm	>450	>400	>300	<300
**ABCD grading system**					
Anterior radius of curvature in 3.0 mm zone centered on thinnest location of cornea	mm	>7.05	>6.35	>6.15	<6.15
Posterior radius of curvature in 3.0 mm zone centered on thinnest location of cornea	mm	>5.70	>5.15	>4.95	<4.95
Thinnest pachymetry, MCT	μm	>450	>400	>300	<300
Distance best-corrected visual acuity		<20/20	<20/40	<20/100	<20/400
	(DEC)	(<1.0)	(<0.5)	(<0.2)	(<0.05)
Slit-lamp biomicroscopy findings		No opacities	No opacities	No opacities	Central opacities
		Vogt's lines	Vogt's lines	Vogt's lines	Vogt's lines
		Cone-shaped cornea	Cone-shaped cornea	Cone-shaped cornea	Cone-shaped cornea

### 3.2 Methods

Keratometry data were collected from Marco ARK-1 Series autorefractor/keratometer. Topography and pachymetry tests were done with WaveLight Oculyzer II corneal topographer. The diagnostic device also works as an optical pachymeter which determines how thick the cornea is. The method does not require contact as it uses light-based technologies to measure the corneal thickness. Furthermore, the application of the technique is painless and highly informative. The results of the pachymetry test are detailed maps of the cornea.

The topography examination provided us with refractometry indices and elevation back parameters obtained from the corneal apex. The best-fit sphere (BFS) is the most common reference for corneal elevation. The sphere has an “exclusion zone”, i.e., a 4.00 mm circle area around the MCT point. The surface area outside the zone is called the “exclusion map”. The raw data of the map are used to compute the elevation back map parameters (50).

Visual acuity was measured with the Golovin-Sivtsev scale, a standardized assessment tool in the countries that use the Cyrillic alphabet (51). The scale scores range from 0.1 to 2.0 decimal units, with 1.0 or 100% for the average vision. A score below 1 indicates myopia, and over 1 suggests far-sightedness or hypermetropia.

#### 3.2.1 Indications for corneal collagen cross-linking

In this study, physicians recommended CXL for patients with confirmed KC progression. The criteria for the progression were as follows: an increase in Kmax of at least 1.0 dioptre (D) over a 6- to 12-month period, a rise in the difference between steep and flat keratometry of ≥1.0 D within 1 year, an increase in average keratometry of ≥0.75 D, a reduction in CCT of ≥2%, a decline in spherical equivalent of more than 0.5 D, and a decrease in uncorrected visual acuity (UCVA) of at least one line on the Snellen chart over 12 months. The last criterion corresponds to a loss of visual acuity significant enough to necessitate new contact lenses more than once every 2 years.

#### 3.2.2 Protocol of corneal collagen cross-linking

We adhered to the standard Dresden Protocol, as G. Wollensak et al. outlined in 2003 (52). Following this protocol, the corneal epithelium in the central area (7–9 mm) was removed and left for 30 min. A 0.1% riboflavin solution mixed with 20% dextran was then applied to the corneal surface. Over the next 30 min, the cornea was irradiated with ultraviolet light at a wavelength of 365–370 nm and an irradiance of 3 mW/cm^2^. Riboflavin was reapplied every 5 min during this period. Throughout the procedure, the cornea absorbed ultraviolet radiation, achieving an energy density of 5.4 J/cm^2^.

We did not study outcomes in patients with very thin cornea. Historically, a corneal thickness of less than 400 μm after removing the epithelium was regarded as a contraindication for CXL. However, the introduction of hypo-osmolar riboflavin solutions allowed for corneal swelling to be induced during the procedure. This swelling increases the corneal thickness before ultraviolet exposure. If the cornea measures less than 400 microns after epithelial removal, the hypotonic riboflavin solution helps elevate the thickness to above 400 microns, making the treatment feasible (53). As a result, studies on hypo-osmolar riboflavin solutions indicated that an excessively thin cornea might no longer pose a limitation for treatment (54, 55).

### 3.3 Study methodology

#### 3.3.1 Data preprocessing and exploration analysis

Upon examination, the study dataset was complete for most variables with few missing values in visiometry findings. Exploratory analysis indicated a strong association between visiometry data and results in pachymetry tests. The relationships were well-defined and consistent, which ensured the statistical robustness of predicting missing values in a linear imputation technique. Linear regression imputation preserved the natural relationships between variables in the dataset, ensuring that the imputed values align with the observed data structure. The exploratory analysis of the dataset followed data preprocessing.

The study cohort was monitored for up to 50 months after CXL. To assess CXL outcomes, we calculated descriptive statistics and then applied the Kruskal–Wallis test to find marked changes in the parameters that did not follow the normal distribution. We resorted to the Student's t-test to compare the normally distributed pre- and postoperative findings. The individual results outside the diapason [15, 85] percentile were considered outliers and excluded from further analysis. The Cohen's D test revealed if the postoperative changes in CCT and MCT were clinically meaningful.

#### 3.3.2 Statistical approaches to tasks

*To complete the first task*, we examined the relationships between CCT and MCT before and after CXL. We used the Shapiro–Wilk test to check the distribution of the data. The relationships between normally distributed variables were tested with Pearson's correlation. For the other variables, the Spearman correlation was used. The significance level of *p* < 0.05 indicated a strong association between the data and CXL outcomes.

*Working on the second task*, we applied the same approach. Specifically, we analyzed associations between pre- and postoperative findings. For this purpose, we computed pairwise correlation coefficients among corneal thickness values, keratometry, topography, visiometry, and refractometry readings.

*To address the third task*, we trained models to predict MCT and CCT changes after CXL from individual preoperative parameters. We selected linear and polynomial equations to model the trends in corneal thickness during the follow-up. Then, we used the Bayesian information criterion (BIC) to score and select the optimal model type.

*To forecast the success of treatment*, we constructed ML models predicting CXL outcomes from the preoperative findings. In the follow-up examinations, corneal thickness reflected the structural outcome of CXL. Therefore, the targeted variables were change in CCT and variation in MCT. Regression models were trained to forecast CCT and MCT dynamics from the four groups of predictors: keratometry readings, visiometry and refractometry data, topography and deviation indices. We performed feature selection and split the original dataset into training and testing subsets - 70 vs. 30% of the data correspondingly.

To train and validate multiple ML models, we used a 5-fold cross-validation technique. Training and validation were conducted with Decision Tree, Random Forest, XGBoost, and LightGBM regressors. The performance was assessed with the mean absolute error (MAE), proportion of MAE to the range of values (ROV), root mean square error (RMSE), and R^2^.

To streamline the training process and augment the models' performance, we implemented Optuna framework and optimized hyperparameters. The framework allowed us to investigate various permutations of hyperparameters for each model. The results of validation tests revealed the top-performing configuration. We measured the models' generalization on unseen data.

## 4 Results

The study showed a significant improvement in keratometry readings and topographic indices after CXL (see Table 2). In visiometry findings, UCVA increased from 0.27 ± 0.23 to 0.33 ± 0.30 (*p* = 0.0286). However, other functional parameters remained stable after the invasion. In early-to-moderate KC cases, UCVA often improves more noticeably because CXL primarily addresses the biomechanical and structural irregularities of the cornea rather than directly altering refractive power (19, 56, 57)

**Table 2:** Characteristics of study participants before and after CXL (mean follow-up period 14 months).

**Parameter (Acronym)**	**Unit**	**Before CXL**	**After CXL**	***p*-value**
**Visiometry, refractometry**				
Sphere refraction	D	-3.06 ± 3.93	-3.63 ± 4.28	0.1891
Axis refraction	°	83.65 ± 49.92	94.14 ± 55.22	0.1057
Uncorrected visual acuity (UCVA)	DEC	0.27 ± 0.23	0.33 ± 0.3	**0.0286**
Corrected (sphere) visual acuity	D	-2.80 ± 3.54	-3.12 ± 3.09	0.3461
Corrected (cylinder) visual acuity	D	-3.49 ± 2.37	-2.98 ± 2.46	0.0744
Corrected (axis) visual acuity	°	90.41 ± 42.63	93.34 ± 48.08	0.5946
Best-corrected visual acuity (BCVA)	DEC	0.62 ± 0.25	0.62 ± 0.26	0.9995
**Pachymetry**				
Minimal corneal thickness (MCT)	μm	457.74 ± 35.56	442.15 ± 40.84	**<0.0001**
Central corneal thickness (CCT)	μm	479.21 ± 38.35	465.57 ± 42.36	**<0.0001**
**Keratometry**				
Corneal astigmatism (Ast)	D	-1.79 ± 3.96	-1.68 ± 3.81	0.6398
Flat corneal curvature (K1)	D	45.64 ± 3.83	44.96 ± 4.03	**<0.0001**
Steep corneal curvature (K2)	D	49.08 ± 4.54	48.61 ± 4.58	**0.0082**
Maximal corneal curvature (Kmax)	D	56.68 ± 6.44	55.61 ± 6.5	**<0.0001**
Radius of K1 (Rf)	mm	7.45 ± 0.60	7.57 ± 0.63	**<0.0001**
Radius of K2 (Rs)	mm	6.90 ± 0.60	6.99 ± 0.65	**0.0047**
Radius of Kmax (Rm)	mm	7.15 ± 0.59	7.26 ± 0.61	**0.0002**
Eccentricity of the cornea (Ecc)	–	0.81 ± 0.41	0.71 ± 0.47	**0.0004**
Average radius of curvature (Rper)	mm	8.01 ± 0.42	9.35 ± 13.1	0.2867
Smallest radius of curvature (Rmin)	mm	6.02 ± 0.66	6.13 ± 0.68	**0.0009**
**Topographic indices**				
Index of surface variance (ISV)	–	98.15 ± 36.72	93.21 ± 38.53	**0.0166**
Index of vertical asymmetry (IVA)	–	1.10 ± 0.46	1.05 ± 0.51	**0.0386**
Keratoconus index (KI)	–	1.27 ± 0.11	1.25 ± 0.13	**0.0154**
Central keratoconus index (CKI)	–	1.07 ± 0.06	1.05 ± 0.08	**0.0039**
Index of height asymmetry (IHA)	–	31.22 ± 27.51	33.49 ± 33.76	0.5460
Index of height decentration (IHD)	–	0.15 ± 0.07	0.14 ± 0.07	**0.0012**
**Belin/Ambrósio deviation indices**				
SD of changes in the front elevation (Df)	–	11.58 ± 6.33	10.17 ± 6.74	**<0.0001**
SD of changes in the back elevation (Db)	–	9.28 ± 5.42	9.42 ± 5.72	0.6484
SD of pachymetric progression (Dp)	–	9.85 ± 5.04	12.83 ± 6.7	**<0.0001**
SD of thinnest point thickness (Dt)	–	2.83 ± 1.44	3.54 ± 1.75	**<0.0001**
SD of thinnest point displacement (Da)	–	3.26 ± 0.68	3.37 ± 0.57	0.0932
Complex index (D)	–	9.50 ± 3.45	9.86 ± 4.01	0.0770

Data are expressed as mean ± SD. Numbers marked in bold indicate statistically significant difference (p < 0.05).

### 4.1 Pre and postoperative pachymetry findings

In our study, the average MCT before CXL was 457.74 ± 35.56 μm. Most protocols for standard epithelium-off CXL require a minimum corneal thickness of 400 μm after epithelial removal. Modified techniques (e.g., hypo-osmolar riboflavin or epithelium-on CXL) can accommodate thinner corneas. In advanced cases, MCT helps determine suitability for other interventions such as corneal transplant or intracorneal ring segment implantation (58).

The average preoperative value for CCT was 479.21 ± 38.35 μm. Although CCT is a hallmark of KC, it does not reflect the clinical stage according to the Amsler-Krumeich classification. MCT shows the disease stage more accurately because the cornea thins in a peripheral or paracentral location, not necessarily at its geometric center. Hence, CCT measurements may miss the area of maximum thinning, leading to an underestimation of disease severity. MCT directly captures the thinnest point of the cornea, providing a more accurate assessment (59, 60).

The average duration of the follow-up period was 14.01 ± 9.98 months. At this time, both CCT and MCT markedly decreased. The thickness of the central cornea dropped from 479.21 ± 38.35 to 465.57 ± 42.36 μm, and MCT reduced from 457.74 ± 35.56 to 442.15 ± 40.84 μm (p < 0.0001) (see Table 2). Transient thinning is a normal response to the treatment. The reasons behind the thinning are dehydration during surgery, keratocyte apoptosis, corneal healing, and remodeling.

According to the Cohen's D test, the postoperative change in MCT was clinically relevant. The test result 0.4758 was approximately 0.5 indicating a moderate magnitude of change. For CCT, the Cohen's D was 0.33. While statistically significant, this change represents a small-to-moderate effect size, which may or may not meet thresholds for clinical relevance depending on specific clinical guidelines or patient outcomes. Hence, the postoperative changes in MCT should serve as the major clinical marker of corneal thinning and thickening since they are more pronounced and meaningful than the CCT dynamics.

An association between MCT and CCT was stronger after CXL (r = 0.9 vs. 0.79, *p* < 0.001). The treatment induces cross-linking uniformly across the corneal stroma, leading to a more homogeneous distribution of biomechanical strength. This uniformity reduces localized thinning and protrusion, resulting in a more consistent relationship between MCT and CCT post-procedure (61).

**Figure 1:**
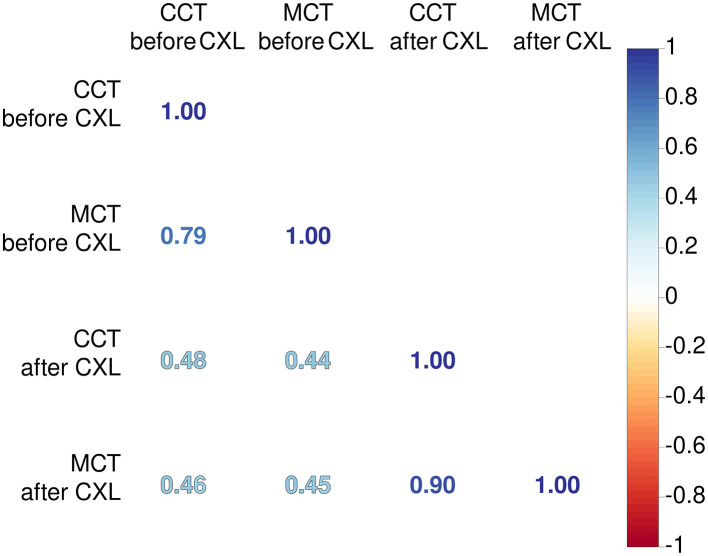
Coefficients of Pearson's correlations between corneal thickness before and after CXL. Color of numbers encodes *p*-level, and all associations are significant with *p* < 0.05.

We also explored relationships between preoperative pachymetry parameters and postoperative findings (see Figure 1). The preoperative MCT and CCT had a weak correlation with pachymetry data after CXL. This suggests the absence of a linear association between preoperative pachymetry data and the structural outcomes of CXL.

### 4.2 Relationship between preoperative parameters and corneal thickness after CXL

**Figure 2:**
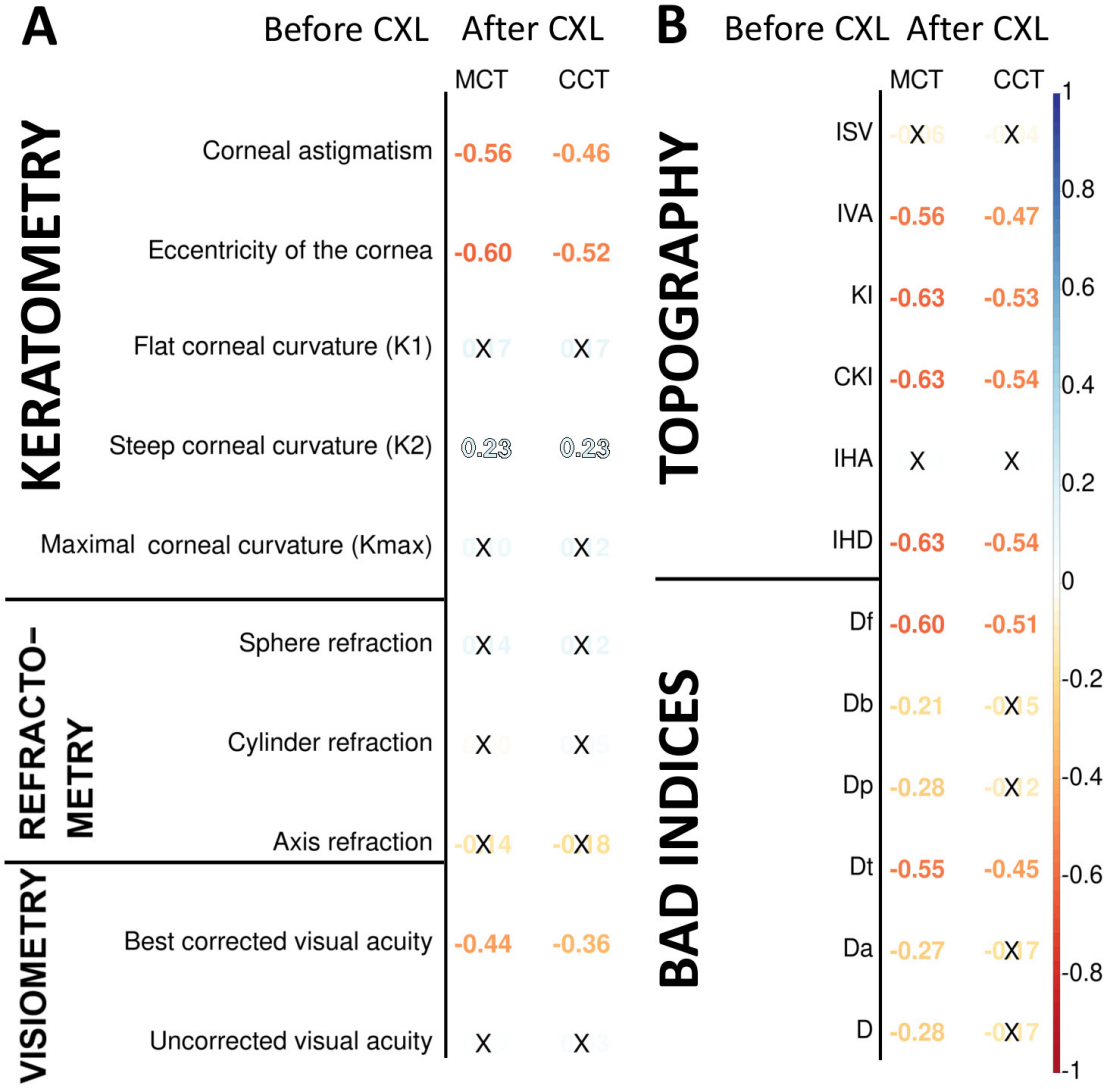
Pearson's correlations of postoperative corneal thickness with keratometry, pachymetry, visiometry, and refractometry findings before CXL **(A)**, corneal topography and deviation indices before CXL **(B)**. Color of numbers encodes *p*-level, and insignificant associations are marked with crosses.

Preoperative keratometry findings exhibited moderate and weak associations with postoperative pachymetry data (see Figure 2A). Corneal eccentricity (Ecc) describes the rate of flattening from the center to the periphery of the cornea, and in our study, it demonstrated a strong inverse correlation with CCT and MCT after CXL (r = −0.52 and −0.60, respectively; *p* = 2.90 × 10^−9^ and *p* = 2.05 × 10^−12^). In analogous to this, corneal astigmatism (Ast) before CXL had a negative correlation with both CCT and MCT after the invasion (r = −0.46, *p* = 2.70 × 10^−7^ and r = −0.56, *p* = 1.51 × 10^−10^ respectively). Higher Ecc and Ast indicate a more advanced disease when the corneal thinning is more severe both before and after the intervention.

The preoperative K2 value had a weak positive correlation with the pachymetry data after CXL: r = 0.23, *p* = 0.001 for both minimal and central corneal thickness. The flat and maximal keratometry readings also had a direct association with the corneal thickness, although it was unremarkable; r = 0.10÷0.17; *p* > 0.05. Before the intervention, corneas with higher keratometry values generally exhibit more advanced ectasia. In these cases, the corneal regions are thinner at or near the cone apex. The biomechanical stability of these corneas is lower, which may influence the remodeling of their tissue in response to CXL. Below is why the connection of postoperative CCT and MCT with K1 and Kmax is slacker than with K2. In KC, the cone is often asymmetric, with the steepest curvature (K2) located away from the central cornea. The post-CXL remodeling is driven by the cone's location and steepness, which are more directly related to K2 than other keratometry values. This suggests a tighter relationship between the preoperative K2 value and pachymetry readings.

Most preoperative functional parameters were weak correlates of the postoperative pachymetry values. An exception was BCVA which exhibited a moderate negative association with MCT (r = −0.44, *p* = 1.31 × 10^−6^). In advanced KC, the cornea is thin and weak before the surgery, which results in worse preoperative BCVA. After CXL, the weak cornea undergoes significant collagen compaction and structural remodeling (15, 62).

Pachymetry data correlated stronger with topography and BAD indices than keratometry and refractometry data (see Figure 2B). All these measurements reflect the structural and geometric properties of the cornea, but topographic and BAD indices are more directly tied to corneal thickness variations (63). The index of height decentration (IHD) directly quantifies the asymmetry of the posterior corneal surface elevation, which is closely linked to the location and severity of corneal thinning in KC (64, 65). For this reason, corneal thickness had a strong negative relationship with IHD (r = −0.63, *p* = 1.31 x 10^−13^).

All topographic readings and the elevation back map parameters correlated stronger with MCT than CCT because MCT provides a more direct measure of the corneal region most affected by ectatic changes in keratoconus. The KI index showed the strongest negative relationship with MCT (r = −0.63, *p* = 1.72 × 10^−13^). The CKI index was associated slightly stronger with both MCT and CCT (r = −0.63, *p* = 1.44 × 10^−13^ and r = −0.54, *p* = 7.56 × 10^−10^, respectively). KI measures the ratio between mean radius values in the upper half and lower half of the cornea. CKI evaluates the ratio between the mean radius of curvature in a peripheral placido ring and the mean radius of curvature of the central ring. These indices are strongly influenced by corneal steepening and asymmetry (40, 66).

**Figure 3:**
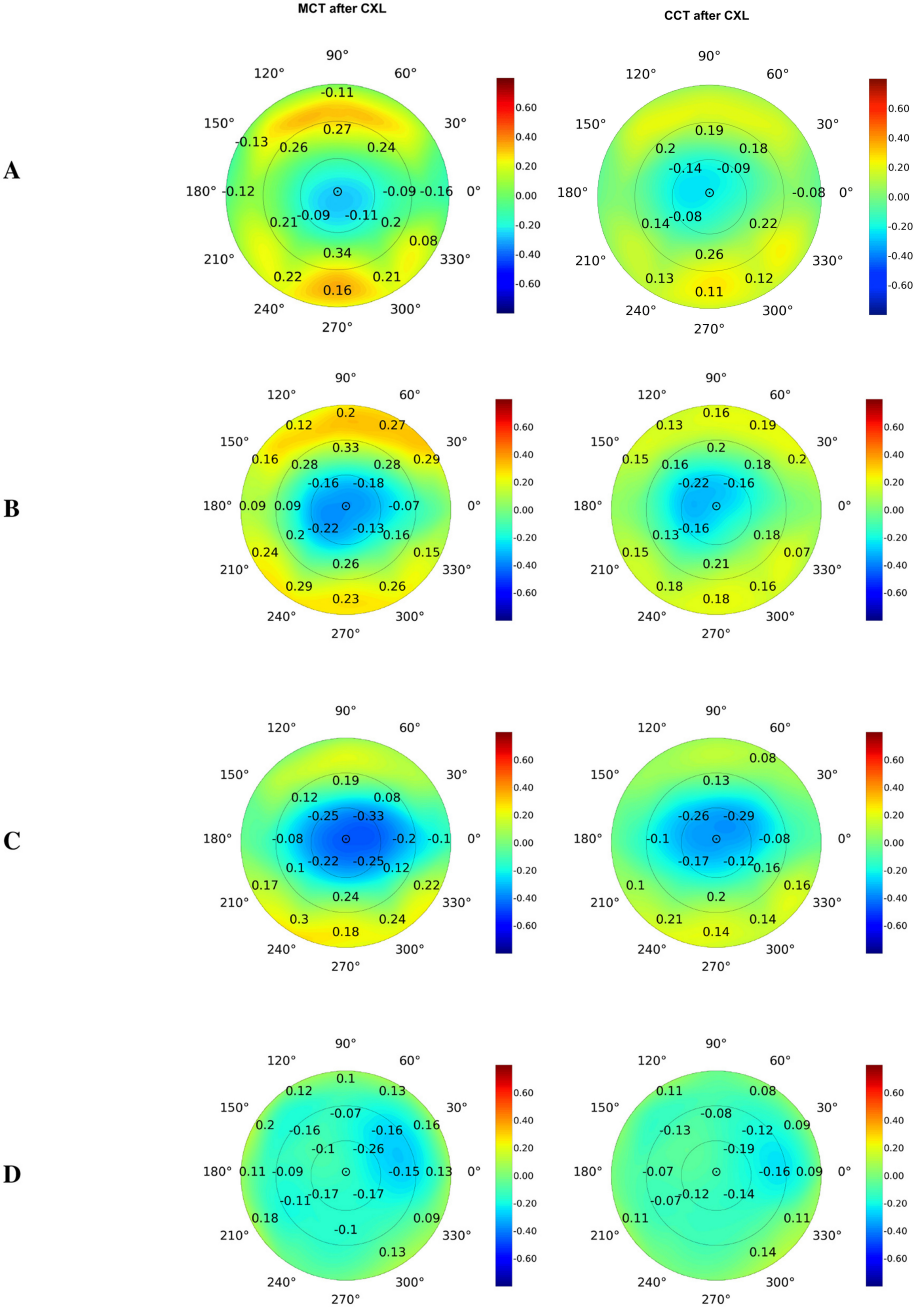
Pearson's correlations of postoperative corneal thickness with preoperative parameters of elevation back maps: posterior elevation **(A)**, posterior elevation with the best-fit sphere **(B)**, exclusion map **(C)**, and exclusion zone **(D)**. Numbers show correlation coefficients for significant associations with *p* < 0.05.

We also studied the correlation of postoperative pachymetry findings with parameters of elevation maps that measure corneal height in micrometers. Posterior elevation represents the true deformation of the cornea. The top correlates of MCT were the data received at the following maps: posterior elevation and posterior elevation with BFS (see Figures 3A, B, respectively). In advanced KC, the cornea is thinner and weaker; therefore, it bulges more at the back.

The exclusion map identifies areas of the cornea that deviate significantly from a reference geometry (67). In our study, the exclusion map area parameters expressed a moderate association with MCT and CCT (see Figure 3C). Meanwhile, findings of exclusion zone showed a weaker correlation with pachymetry measurements (see Figure 3D). In KC, structural changes in the cornea directly impact the exclusion map data. The exclusion zone parameters capture broad geometric characteristics of the cornea, and they reflect the localized corneal thinning less reliably (67).

### 4.3 Corneal pachymetry after CXL

**Figure 4:**
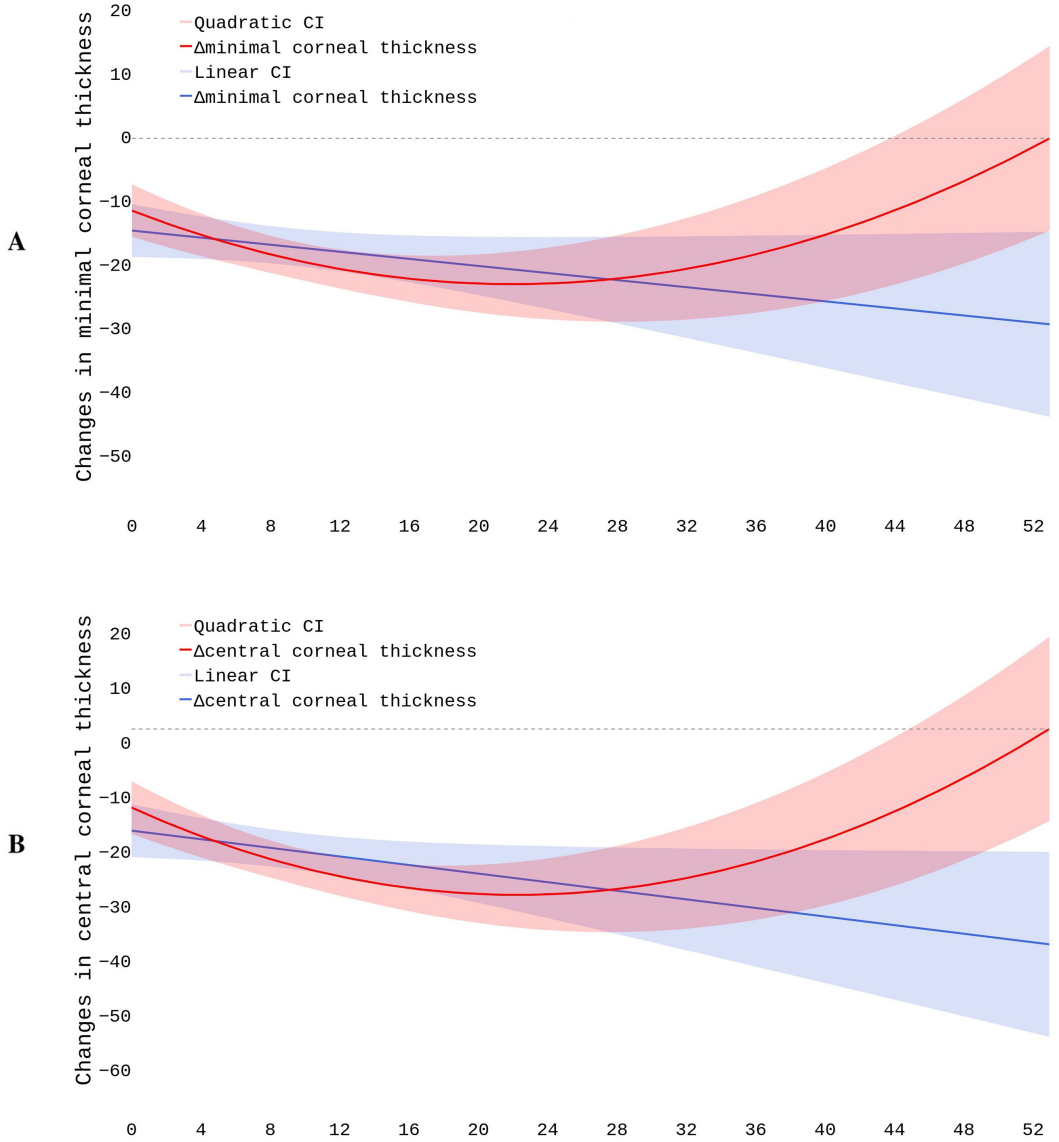
Postoperative changes in minimal **(A)** and central **(B)** corneal thickness.

We modeled postoperative structural changes with linear and polynomial regressions. The dataset included 796 cases. The observational period length ranged from 4 to 52 months. As seen in Figure 4, the linear trends showed a steady decline in pachymetry readings. The slope describing the MCT dynamics was negative but non-significant: β = −0.1891, *p* = 0.169 (see Figure 4A). The polynomial function depicting the relationship between time and change in MCT also declined during the first 20 months and then reached a plateau. The trend exhibited a positive shift starting from the 28th month of observation, with a reversal toward baseline MCT 52 months after the surgery. The further prognosis might be inaccurate because our study lacks observations covering the later stages of recovery. The BIC value was slightly lower in the first- than in the second-degree equation: 1692.6 vs. 1697.3, respectively. Although this fact suggested a linear dependency between MCT and time, the MAE values for the first- and second-degree equations were almost equal: 21.68 vs. 21.71 μm.

The plot describing residual vs. fitted values indicated that the linear model did not accommodate the data properly, and the polynomial model match them slightly better. These findings suggest a non-linear association between time after surgery and postoperative MCT values. Still, the structured residuals indicate that other factors may account for the large variance in corneal thickness after CXL. Q-Q plots revealed that residuals did not follow the normal distribution. This could indicate the presence of outliers or non-constant variance impacting model's reliability. In clinical settings, this suggests including other predictors in addition to the duration of observation (see Section 4.4).

For dynamics in CCT, the slope coefficient was negative and significant: β = −0.3002, *p* = 0.035 (see Figure 4B). The second-degree polynomial model of changes in CCT also reflected the postoperative dynamics more accurately than the linear regression. The BIC and MAE for these models were close: 1697 vs. 1701 and 22.50 vs. 22.38 μm, respectively. In the plot describing residual vs. fitted values, residuals followed a more structured pattern in the polynomial than linear regression. This justified the non-linear relationship between the time and change in CCT. Q-Q plots showed a significant deviation of residuals from the normal distribution both in the linear and quadratic equations.

The quadratic function drew a U-shaped trend in CCT changes during the first 4.3 years after CXL. In the first 20 months, the thinning was pronounced. Then, the corneal thickness remained stable for approximately 8 months. Between the 28th and 52nd months, the cornea progressively recovered its baseline thickness. Afterwards, the cornea continued thickening. The postoperative variation in pachymetry tests largely depends on the characteristics of the study cohort. In the early stages of the disease, the cornea's potential to recover its thickness after CXL is largely higher than in advanced KC.

The reported R^2^ values indicate extremely poor model fit. It was 0.0006 vs. 0.0007 and 0.0005 vs. 0.0012 for the linear and polynomial functions describing MCT and CCT models, respectively. Hence, neither regression model effectively predicts postoperative data. The findings suggest that unaccounted factors affect the postoperative fluctuations in the pachymetry data. These factors may include biomechanical response variability after CXL, patient-specific healing mechanisms, corneal hydration, and epithelial remodeling. To improve the accuracy of the prediction, we included preoperative findings in the models.

### 4.4 Prognosing CXL outcomes in KC patients

In our models, the prediction of postoperative MCT variations was more accurate than CCT. For a reliable prognosis of postoperative corneal thickness, we used several groups of findings: keratometry readings, visiometry and refractometry data, topography and BAD indices. We also investigated whether the prediction accuracy improves with the top correlating features from various modalities at the input to the model.

**Figure 5:**
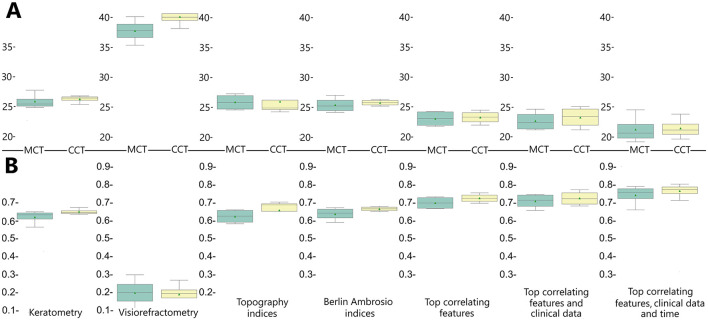
Accuracy of regression models trained to predict postoperative changes in minimal and central corneal thickness reported in R^2^ **(A)** and RMSE **(B)**.

Combinations of the top correlates with clinical data and time after surgery showed the highest predictive potential (see Tables 3, 4, Figure 5). The reliability of the models trained to predict MCT from preoperative findings was high: R^2^ = 0.71 ± 0.02 and RMSE = 22.71 ± 0.60. The performance metrics for CCT models were 0.73 ± 0.01 and 23.29 ± 0.23. Hence, risk assessment is more accurate with multimodal preoperative diagnostics than unimodal.

**Table 3:** Regression metrics for predicting *minimal corneal thickness*.

**Diagnostic modality**	**Predictors**	**Mean SHAP**	**Model**	**RMSE _*train*_**	**RMSE _*test*_**	**R^2^_*train*_**	**R^2^_*test*_**	**MAE/ROV _*train*_,%**	**MAE/ROV _*test*_,%**
Keratometry	Ast, Rf, Rs, Rm, Rper, Ecc, Axis up, Axis low, Rmin	1.62	DT	23.04 ± 0.22	24.98 ± 0.08	0.6997 ± 0.0058	0.6509 ± 0.0024	6.08 ± 0.05	6.27 ± 0.02
			RF	18.12 ± 0.49	25.26 ± 0.09	0.8141 ± 0.0101	0.6431 ± 0.0026	4.13 ± 0.15	5.47 ± 0.08
			XGB	15.59 ± 0.17	27.86 ± 0.24	0.8625 ± 0.0031	0.5656 ± 0.0074	3.06 ± 0.16	5.87 ± 0.03
			LGBM	17.60 ± 0.50	25.85 ± 0.20	0.8247 ± 0.0099	0.6261 ± 0.0057	4.17 ± 0.19	5.77 ± 0.10
Visiometry, refractometry	Sphere refraction, Axis refraction, UCVA, CVA sphere, CVA cylinder, CVA axis, BCVA	0.97	DT	34.48 ± 0.66	38.48 ± 0.41	0.3272 ± 0.0257	0.1718 ± 0.0178	9.66 ± 0.14	10.94 ± 0.03
			RF	21.65 ± 0.71	35.36 ± 0.12	0.7347 ± 0.0174	0.3003 ± 0.0049	5.98 ± 0.22	9.84 ± 0.05
			XGB	12.40 ± 3.35	40.16 ± 0.74	0.9066 ± 0.0581	0.0975 ± 0.0329	1.88 ± 1.46	10.75 ± 0.04
			LGBM	24.80 ± 3.59	37.13 ± 0.30	0.6449 ± 0.1041	0.2287 ± 0.0125	6.74 ± 1.10	10.41 ± 0.13
Topographic indices	ISV, IVA, KI, CKI, IHA, IHD	0.76	DT	22.36 ± 0.56	27.26 ± 0.48	0.7169 ± 0.0143	0.5842 ± 0.0147	5.84 ± 0.18	6.34 ± 0.13
			RF	17.87 ± 0.13	24.74 ± 0.03	0.8193 ± 0.0027	0.6574 ± 0.0009	3.97 ± 0.06	5.36 ± 0.02
			XGB	15.76 ± 0.12	26.85 ± 0.45	0.8596 ± 0.0021	0.5965 ± 0.0136	3.15 ± 0.12	5.72 ± 0.04
			LGBM	17.96 ± 1.13	24.56 ± 0.35	0.8169 ± 0.0242	0.6624 ± 0.0098	4.24 ± 0.36	5.62 ± 0.14
BAD indices	Df, Db, Dp, Dt, Da, D	4.00	DT	19.53 ± 0.70	26.99 ± 0.18	0.7839 ± 0.0163	0.5924 ± 0.0054	4.94 ± 0.18	5.85 ± 0.03
			RF	16.60 ± 0.16	24.14 ± 0.08	0.8440 ± 0.0031	0.6740 ± 0.0023	3.65 ± 0.05	4.89 ± 0.04
			XGB	14.93 ± 0.04	25.88 ± 0.12	0.8739 ± 0.0006	0.6254 ± 0.0035	2.90 ± 0.05	5.25 ± 0.01
			LGBM	17.40 ± 1.06	24.56 ± 0.23	0.8282 ± 0.0215	0.6625 ± 0.0063	4.11 ± 0.29	5.29 ± 0.12
**Multimodal models**									
Top correlating features	Ecc, BCVA, CKI, Df, Dt, Rf, Rs, Rper, IVA, KI, IHD	3.51	DT	17.28 ± 0.94	24.20 ± 0.80	0.8307 ± 0.0195	0.6721 ± 0.0207	4.52 ± 0.25	5.23 ± 0.07
			RF	14.80 ± 0.54	21.80 ± 0.10	0.8760 ± 0.0091	0.7340 ± 0.0025	3.38 ± 0.10	4.49 ± 0.04
			XGB	10.71 ± 0.34	24.30 ± 0.54	0.9351 ± 0.0042	0.6695 ± 0.0147	1.68 ± 0.29	4.80 ± 0.02
			LGBM	15.87 ± 1.44	22.05 ± 0.43	0.8564 ± 0.0266	0.7280 ± 0.0106	3.78 ± 0.39	4.94 ± 0.10
Top correlating features and clinical data	Top features and Sex, Age after surgery, Bilateral pathology, Clinical stage	1.91	DT	18.56 ± 1.96	23.50 ± 1.56	0.8030 ± 0.0404	0.6895 ± 0.0417	4.89 ± 0.48	5.43 ± 0.05
			RF	13.79 ± 0.21	21.44 ± 0.04	0.8925 ± 0.0033	0.7428 ± 0.0009	3.17 ± 0.06	4.40 ± 0.02
			XGB	7.97 ± 0.77	24.68 ± 0.62	0.9637 ± 0.0076	0.6591 ± 0.0170	1.43 ± 0.37	4.98 ± 0.04
			LGBM	14.51 ± 1.30	21.22 ± 0.19	0.8799 ± 0.0208	0.7480 ± 0.0045	3.48 ± 0.25	4.73 ± 0.05
Top correlating features, clinical data and time	Previous group and Time after surgery	2.55	DT	15.41 ± 0.61	24.57 ± 0.16	0.8654 ± 0.0110	0.6620 ± 0.0045	4.12 ± 0.18	5.09 ± 0.11
			RF	10.09 ± 0.62	20.05 ± 0.16	0.9422 ± 0.0071	0.7750 ± 0.0036	2.49 ± 0.11	4.17 ± 0.04
			XGB	1.58 ± 0.52	21.32 ± 0.13	0.9984 ± 0.0012	0.7456 ± 0.0032	0.26 ± 0.16	3.85 ± 0.02
			LGBM	11.20 ± 0.72	19.22 ± 0.36	0.9287 ± 0.0090	0.7932 ± 0.0079	2.70 ± 0.15	4.35 ± 0.11

CVA, corrected visual acuity; DT, Decision Tree regressor; LGBM, Light Gradient Boosting Machine; R, radius of curvature; RF, Random Forest Regressor; XGB, XGBoost.

RMSE (train and test) and R^2^ (train and test) are reported as mean ± standard deviation for each model.

**Table 4:** Regression metrics for predicting *central corneal thickness*.

**Diagnostic modality**	**Predictors**	**Mean SHAP**	**Model**	**RMSE _*train*_**	**RMSE _*test*_**	**R^2^_*train*_**	**R^2^_*test*_**	**MAE/ROV _*train*_,%**	**MAE/ROV _*test*_,%**
Keratometry	Ast, Rf, Rs, Rm, Rper, Ecc, Axis up, Axis low, Rmin	1.38	DT	24.14 ± 0.25	26.90 ± 0.19	0.7096 ± 0.0061	0.6371 ± 0.0053	6.48 ± 0.07	7.06 ± 0.04
			RF	19.26 ± 0.20	25.45 ± 0.05	0.815 ± 0.0038	0.6752 ± 0.0013	4.74 ± 0.05	6.26 ± 0.04
			XGB	16.81 ± 0.20	26.66 ± 0.20	0.8592 ± 0.0034	0.6435 ± 0.0055	3.66 ± 0.21	6.43 ± 0.04
			LGBM	18.98 ± 0.43	26.39 ± 0.19	0.8204 ± 0.0084	0.6507 ± 0.0051	4.84 ± 0.16	6.60 ± 0.08
Visiometry, refractometry	Sphere refraction, Axis refraction, UCVA, CVA sphere, CVA cylinder, CVA axis, BCVA	1.04	DT	36.71 ± 0.79	42.31 ± 0.08	0.3283 ± 0.0295	0.1026 ± 0.0037	11.1 ± 0.24	12.6 ± 0.03
			RF	25.73 ± 0.78	38.17 ± 0.15	0.6698 ± 0.0201	0.2697 ± 0.0059	7.47 ± 0.23	11.3 ± 0.05
			XGB	11.45 ± 0.29	40.00 ± 0.31	0.9346 ± 0.0035	0.1978 ± 0.0128	1.45 ± 0.31	11.5 ± 0.11
			LGBM	25.87 ± 2.62	40.09 ± 0.46	0.6632 ± 0.0684	0.1941 ± 0.0185	7.45 ± 0.82	11.9 ± 0.15
Topographic indices	ISV, IVA, KI, CKI, IHA, IHD	0.89	DT	23.17 ± 0.89	29.82 ± 0.77	0.7321 ± 0.0213	0.5538 ± 0.0229	6.18 ± 0.27	7.76 ± 0.22
			RF	18.95 ± 0.40	24.24 ± 0.11	0.8209 ± 0.0076	0.7055 ± 0.0029	4.67 ± 0.07	5.93 ± 0.04
			XGB	17.02 ± 0.15	24.98 ± 0.31	0.8557 ± 0.0026	0.6871 ± 0.0078	3.79 ± 0.13	6.06 ± 0.04
			LGBM	18.93 ± 0.26	24.76 ± 0.37	0.8213 ± 0.0050	0.6925 ± 0.0092	4.87 ± 0.10	6.26 ± 0.11
BAD indices	Df, Db, Dp, Dt, Da, D	4.41	DT	21.15 ± 0.30	26.35 ± 0.25	0.7771 ± 0.0064	0.6519 ± 0.0066	5.61 ± 0.09	6.59 ± 0.03
			RF	18.45 ± 0.52	25.18 ± 0.20	0.8303 ± 0.0098	0.6820 ± 0.0053	4.55 ± 0.17	5.85 ± 0.07
			XGB	16.36 ± 0.07	26.03 ± 0.14	0.8666 ± 0.0012	0.6601 ± 0.0037	3.56 ± 0.08	6.06 ± 0.02
			LGBM	18.35 ± 0.83	25.45 ± 0.34	0.8318 ± 0.0158	0.6751 ± 0.0088	4.70 ± 0.31	6.13 ± 0.09
**Multimodal models**									
Top correlating features	Ecc, BCVA, CKI, Df, Dt, Rf, Rs, Rper, IVA, KI, IHD	3.49	DT	20.89 ± 0.45	24.51 ± 0.46	0.7824 ± 0.0092	0.6988 ± 0.0116	5.78 ± 0.07	6.22 ± 0.06
			RF	16.52 ± 0.18	22.76 ± 0.05	0.864 ± 0.0031	0.7402 ± 0.0013	4.08 ± 0.07	5.37 ± 0.02
			XGB	12.50 ± 0.13	23.93 ± 0.11	0.9221 ± 0.0017	0.7129 ± 0.0027	2.03 ± 0.22	5.8 ± 0.02
			LGBM	16.71 ± 0.74	22.02 ± 0.19	0.8606 ± 0.0122	0.7569 ± 0.0042	4.25 ± 0.20	5.56 ± 0.06
Top correlating features and clinical data	Top features and Sex, Age after surgery, Bilateral pathology, Clinical stage	1.95	DT	18.10 ± 0.55	25.11 ± 0.37	0.8366 ± 0.0104	0.6837 ± 0.0093	5.14 ± 0.12	6.10 ± 0.04
			RF	15.59 ± 0.37	22.27 ± 0.13	0.8788 ± 0.0059	0.7512 ± 0.0031	0.04 ± 0.00	0.05 ± 0.00
			XGB	9.35 ± 0.18	24.56 ± 0.18	0.9564 ± 0.0018	0.6975 ± 0.0045	1.44 ± 0.22	5.86 ± 0.03
			LGBM	15.41 ± 1.15	21.22 ± 0.21	0.8810 ± 0.0184	0.7741 ± 0.0046	4.02 ± 0.30	5.34 ± 0.09
Top correlating features, clinical data and time	Previous group and Time after surgery	2.51	DT	18.04 ± 1.49	23.84 ± 0.44	0.8367 ± 0.0289	0.7148 ± 0.0108	5.21 ± 0.34	6.02 ± 0.16
			RF	12.24 ± 0.66	20.74 ± 0.15	0.9251 ± 0.0081	0.7842 ± 0.0031	3.32 ± 0.15	4.87 ± 0.04
			XGB	1.66 ± 0.00	21.62 ± 0.20	0.9986 ± 0.0012	0.7655 ± 0.0044	0.17 ± 0.01	4.79 ± 0.04
			LGBM	12.32 ± 1.11	19.68 ± 0.34	0.9237 ± 0.0138	0.8056 ± 0.0069	3.29 ± 0.30	4.98 ± 0.16

CVA, corrected visual acuity; DT, Decision Tree regressor; LGBM, Light Gradient Boosting Machine; R, radius of curvature; RF, Random Forest Regressor; XGB, XGBoost.

RMSE (train and test) and R^2^ (train and test) are reported as mean ± standard deviation for each model.

**Figure 6:**
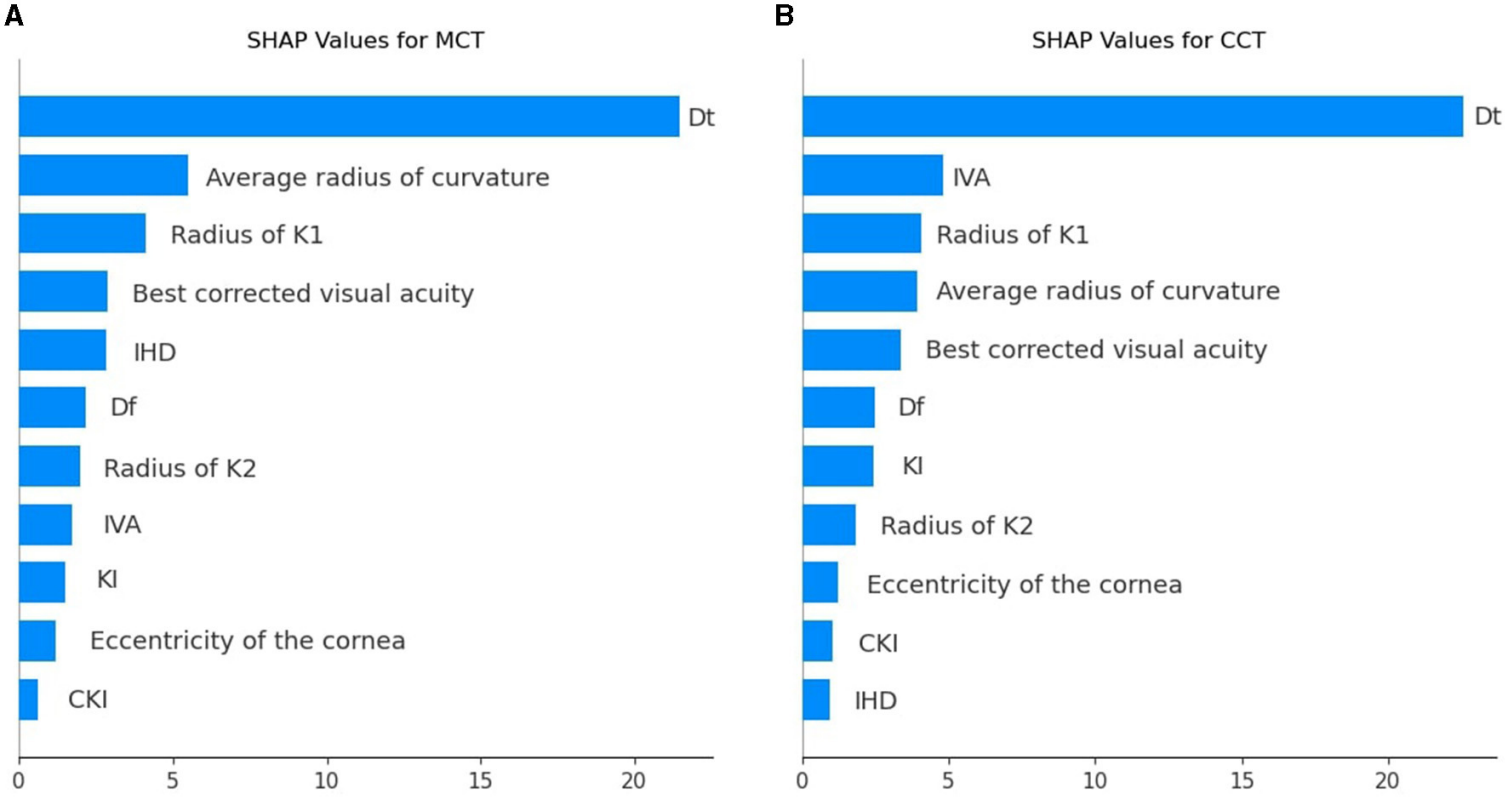
SHAP values for individual predictors among the top correlating features used to prognosticate postoperative MCT **(A)** and CCT **(B)**.

To illustrate the impact of predictors on model outcomes, we computed SHAP values for each variable group and averaged the summary value across individual variables within those groups. In BAD indices, the mean SHAP value was maximal (see Tables 3, 4). However, only BAD-Dt had a strong predictive power since it indicates the thinnest point thickness of the cornea. Many BAD indices had lower predictive power than individual keratometry, visiometry, and topography findings (see Figure 6).

Dt and MCT are often used interchangeably, but they may differ and show a reverse association in certain situations where the thinnest point lies outside the central cornea. In ectatic corneas (e.g., keratoconus), the thinnest point is often displaced and located in the inferior-temporal region. Diagnostic devices can define MCT differently. If MCT is measured only in the central cornea, it may not capture the actual thinnest point outside this region. As a result, MCT in the central zone might appear thicker than Dt, creating a reverse association. A comprehensive analysis of all preoperative findings allows one to assess risks in CXL reliably.

When trained on the findings of each diagnostic modality, the models with BAD indices achieved the top performance: R^2^ = 0.64 ± 0.004 and 0.67 ± 0.01, RMSE = 23.39 ± 0.15 and 25.76 ± 0.24 for MCT and CCT models, respectively. A high SHAP value of Dt serves as an explanation of the high predictive potential of the BAD findings. The predictive potential of topographic findings was slightly lower: R^2^ = 0.63 ± 0.01 and 0.66 ± 0.02, RMSE = 25.85 ± 0.33 and 25.95 ± 0.39, for MCT and CCT prognosis. The pre-operative IHD value was a strong predictor of MCT. IHD captures vertical decentration and corneal asymmetry–the determinants of corneal thinning and remodeling after CXL. The IVA index was the second top informative predictor of CCT. Higher IVA values indicate steeper inferior curvature, which is often associated with corneal thinning in the central regions.

Models trained on keratometry readings were slightly less reliable than those trained on BAD indices: R^2^=0.62 ± 0.004 and 0.65 ± 0.00, RMSE = 25.99 ± 0.15 and 26.36 ± 0.16 for MCT and CCT, respectively. Keratometry findings characterize surface shape but do not reflect how the cornea will respond structurally to CXL. According to our results, visiometry and refractometry data were the worst predictors of postoperative MCT and CCT: R^2^ = 0.20 ± 0.02 and 0.19 ± 0.01, RMSE = 37.78 ± 0.40 and 40.15 ± 0.25, respectively. Vision characteristics and refraction parameters illustrate the optical outcome of the disease. They show a loose correlation with pachymetry data, which explains the questionable performance of the models trained on visiometry and refractometry findings.

## 5 Discussion

### 5.1 Pachymetry after corneal collagen cross-linking

A limited number of studies revealed postoperative changes in MCT and CCT. Meanwhile, monitoring corneal thickness is important because it ensures the absence of complications: corneal ectasia, wound dehiscence, delayed healing, and decreased intraocular pressure (68, 69). Our observations revealed mid-term CXL outcomes (approximately 4 years). The corneal thickness dropped after CXL, and the negative trend continued for approximately 52 months. In some studies, authors followed the patients with mild KC for 12 months, and they also showed a steady decline in corneal thickness (70, 71).

Our findings confirmed the relationship between pre- and postoperative pachymetry values, both central and minimal. Hence, the baseline pachymetry data can reflect the intervention outcomes. In the current study, both preoperative CCT and MCT findings correlated higher with the preoperative CCT than with MCT. These results suggest that researchers should recognize CCT as an important input to models prognosticating results in pachymetry tests after the surgery. Currently, MCT is considered a strong predictor of CXL effectiveness (15, 72).

Preoperative CCT and MCT findings correlated closely (r = 0.79, p < 0.001). In KC, steepening of the cornea is irregular, which results in irregular astigmatism (1). The association between CCT and MCT after CXL strengthened compared to the preoperative status (r = 0.90 vs. 0.79, p < 0.001). Hence, CXL halts the KC progression, and changes in the corneal thickness become more uniform due to a decrease in the interfibrillar distance (73).

A postoperative decline in pachymetry values is a natural process of corneal remodeling after CXL. In our study, the alterations were accompanied by an improvement in UCVA and keratometric values. Other studies also reported an improvement in visual acuity after CXL (74, 75). However, the study authors focused primarily on corrected distance visual acuity. In their works, the functional outcome of CXL depended on preoperative visual parameters (74, 75). In another study, an improvement in vision after CXL did not correlate significantly with the change in corneal thickness (15). Complex variations in the biomechanics of the cornea may account for the discrepant findings in recent studies on the restoration after CXL.

### 5.2 Association between preoperative ophthalmometry findings and CXL outcomes

The corneal refractive power depends on the corneal thickness and curvature (76). The majority of KC studies revealed a change in visual refractometry, keratometry readings, and corneal flattening after CXL (77, 78). Clinical trials should consider the baseline corneal thickness for designing a CXL protocol and prescribing the treatment to the patients because the outcome depends on initial pachymetry findings (79, 80). Previous studies rarely used pachymetry data to evaluate the CXL effectiveness. Keratometry remains the major outcome measurement in the assessment of the disease dynamics because the key factors affecting refraction are the refractive index of the cornea and adjacent tear film (81). A recent publication supports the correlation between the corneal thickness and Kmax (82).

The Pentacam topography screening indices (ISV, IVA, KI, CKI, etc.) form a corneal thickness profile that reflects variations of the thinnest point in the peripheral cornea. According to our data, the indices are the top correlates of MCT post-CXL. From other studies, corneal topography is the best method for early detection and monitoring progression of KC (83).

The current study revealed a negative relationship between postoperative pachymetry findings and preoperative data on BCVA and Ast. The relationship between preoperative visual acuity and postoperative corneal thickness after CXL is not straightforward as these factors are influenced by multiple variables, including the severity of keratoconus, corneal biomechanics, and the CXL procedure itself. A recent study revealed an association between preoperative corneal thickness and postoperative corrected visual acuity (84). Visual acuity is a marker of a functional result of CXL, and corneal thickness depicts structural consequence of the procedure. Functional and structural outcomes of CXL are tightly connected. Still, a confounding factor may account for the loose association between preoperative results in the vision test and postoperative corneal thickness.

Our study showed a pronounced correlation between postoperative corneal thickness and preoperative topographic indices such as IVA, KI, CKI, and IHD. IVA quantifies surface variance and vertical asymmetry, and it might serve as an indirect predictor of corneal biomechanical behavior post-CXL. Greenstein et al. reported an improvement in 3 of 6 topographic indices a year after CXL, suggesting an overall improvement in corneal shape (15). Researchers used topographic findings to develop new treatment methods, such as topography-guided CXL and accelerated CXL combined with topography-guided photorefractive keratectomy (85, 86). However, their findings do not explain the direct correlations of topographic indices with pachymetry changes.

We identified a weak-to-moderate relationship between preoperative elevation back map parameters and the pachymetry findings after CXL. The posterior surface of the cornea has more fluid than the anterior wall, and it is a more sensitive indicator of abnormality (87). Therefore, it was necessary to study the association of the elevation back map parameters with postoperative outcomes. In a study on corneal remodeling, elevation back map values significantly increased during a year after the treatment (88). Still, it is challenging to explain the relationship between preoperative parameters of the posterior surface of the cornea and pachymetry values after CXL.

### 5.3 Long-term changes in corneal curvature after CXL

We used linear and polynomial models to forecast a long-term change in the corneal thickness after CXL. First-degree models showed negative linear trends in CCT and MCT values, which replicates the literature data. Other authors also observed a linear decrease in pachymetry values (16, 89). They reported a pronounced thinning of the cornea in the first year after the intervention (16).

The linear model constructed by us was sensitive to the initial steep decline, and the steady negative tendency in the corneal thickness persisted for 4 years. The recovery phase might be non-uniform across all patients (90), and pachymetry values did not return to the baseline values in all the cases. For this reason, the linear model predicted a decrease even though the thickness stabilized or partially recovered later.

According to our data, the polynomial curve demonstrated the complex time-to-corneal-thickness association more reliably than the linear trendline. The findings reflect the natural course of post-CXL corneal remodeling. It includes thinning immediately after CXL and stabilization with partial recovery of corneal thickness over time. Both MCT and CCT show progressive reduction throughout the first 20 months of observation. Thereafter, corneal thickening occurs, restoring baseline values at ~52 months post-surgery.

Meanwhile, studies by Greenstein et al. showed that the cornea almost regained its minimal thickness in 12 months after CXL (15, 32). In these studies, the cases were more severe than in our observation, which is evident from the higher preoperative Kmax (60.9 ± 9.5 D) and lower MCT (440.7 ± 52.9 μm). Hence, it is not clear what may account for the quick regeneration of the cornea in the report by Greenstein on the efficiency of the standard CXL protocol.

A study by Holopainen et al. also revealed quick positive dynamics in MCT. The authors found that corneal thickness decreased shortly after cross-linking. However, at the follow-up 6 months later, the cornea regained its original thickness (91). Mild severity of the observed cases may explain the rapid improvement in this cohort of patients. In that study, the preoperative value for Kmax was 48.9 ± 3.7 vs. 56.68 ± 6.44 D in our research. Before CXL, the corneas were also markedly thicker: The average MCT was 483 ± 54 vs. 458 ± 36 μm in our observation. The marked difference in the patient populations may rationalize the discrepancies in findings between the studies.

An article by Chan et al. revealed postoperative changes after accelerated CXL. The cases were more severe than in our study: Preoperative Kmax was 61.99 ± 10.37 D. However, the corneal thinning was less pronounced than in our research: MCT before the treatment was 467.05 ± 38.59 μm. According to these authors, MCT slightly decreased to 454.84 ± 47.21 μm 2 years after the intervention, and the cornea continued thinning. In 5 years post-CXL, the size dropped to 452.68 ± 60.12 μm (p < 0.05), and the reduction in MCT became pronounced (89). The postoperative dynamics of this observation was worse than in our research, probably, due to more severe cases and application of another CXL protocol.

Earlier studies reported contradicting findings on the dynamics of corneal thinning after CXL. Differences in patient populations account for heterogeneity in literature data. For example, Holopainen et al. observed corneal thinning within the first month with a gradual thickening within the next 5 months (91). The patients regained the original corneal thickness 6 months after the treatment because they had mild disease forms. In their cases, the preoperative MCT was 483 ± 54 μm, and the Kmax value was 48.9 ± 3.7 D, which corresponds to the 1st stage in the Amsler-Krumeich classification. Greenstein et al. received similar findings. According to them, the cornea thinned at 1 month and from the 1st to the 3rd month. Then, the thickness recovered between the 3rd and the 6th month. Still, it is challenging to interpret these data because the authors reported neither the preoperative Kmax nor the disease stage (15).

Corneal thickness below 400 μm was an exclusion criterion in our research. Patients with skinny corneas (<400 μm) are at risk for excessive corneal thinning and potential complications. In a study with preoperative Kmax values of 61.70 ± 11.10 D (stage 4), patients were followed, on average, for 36 months after the intervention. Despite the lengthy observation, the cornea thinned from 460.9 ± 51.4 to 434.9 ± 63 μm (18). In another study, patients with preoperative MCT lower than 430μm showed early progression after conventional CXL (92). Knowledge on this motivated us to exclude cases with skinny corneas from the research. In skinny corneas, accelerated CXL protocol is safer. Still, the application of this protocol does not ensure the success of the intervention (89).

#### 5.3.1 Changes in biomechanical properties of the cornea after CXL

In the early stages of KC, biomechanical changes are hard to detect and measure *in vivo*. Therefore, researchers use animal models or human eye-banked corneas to study corneal changes (93–95). The refinements of the affected cornea include a reduction in the number of collagen fibril lamellae and a decrease in the density of the sub-basal nerve plexus, endothelial cells, and stromal keratocytes (96–99). CXL stops further corneal weakening by creating new covalent bonds in the corneal stroma (95). The formed cross-links hold collagen fibers (100). Some studies observed an improvement in collagen fibril organization due to CXL (101). The diameter of anterior stromal collagen fibers increases more markedly (102).

Hydration impacts the biomechanical characteristics of the cornea. After CXL, the hydration level rises making the cornea stiffer to stretch (103). An *in vivo* study showed that the cornea thins and loses water after CXL (104, 105). Then, it recovers toward baseline thickness, and the level of hydration restores (106). The endothelium regulates corneal hydration (107). In KC, the endothelial cell loss due to apoptosis alters water-electrolyte balance and distorts the cornea. Although CXL improves corneal function, the procedure possesses side effects. It decreases the endothelial cell density by producing reactive oxygen species, which are cytotoxic in high concentrations (108, 109).

### 5.4 Determinants of CXL effectiveness in KC patients

Information on the determinants of CXL success is limited (110). For prognosticating postoperative outcomes, researchers commonly perform univariate analysis, which is the most straightforward data processing procedure (111). We focused on the structural outcomes of CXL and trained ML algorithms to forecast the postoperative corneal thickness. In both CCT and MCT models, the best predictors were a combination of the top correlates with clinical data and time after surgery. Recent studies also revealed the prediction of CXL efficiency from multiple parameters (112, 113, 169, 170). However, the authors of these studies focused on the CXL's functional outcomes (refractive power). Meanwhile, our research gives a new insight into the structural consequences of the intervention (corneal thinning).

The current research revealed that preoperative BAD indices are the most reliable predictors of postoperative MCT and CCT. Recent studies also suggested these indices as informative predictors of CXL efficiency (113–115). The authors of these studies used functional parameters and keratometry values as the markers of CXL outcomes. In contrast to them, we focused on structural data (results in pachymetry tests).

Keratometry readings and topographic indices are widely used in research to predict CXL outcomes. Our study showed that the predictive potential of these parameters is slightly lower than that of BAD indices. Other authors also consider keratoconus enlargement and preoperative longitudinal changes in corneal topography as prognosing factors of CXL efficiency (83, 116). In our database, most cases had a single baseline examination before the invasion. Therefore, we could not explore longitudinal preoperative findings as potentially informative features. Future studies may improve the prognostication of CXL outcomes with the suggested model inputs.

The visiometry and refractometry findings had the least predictive value in our research. Contrarily, Badawi and Abou Samra showed a strong linear dependency between preoperative BCVA and CXL outcomes (β = −0.945, p < 0.001) (16). In that study, the marker of CXL efficiency was postoperative Kmax, unlike corneal thickness in our study. In untreated KC, a univariate model based on BCVA reveals the disease progression non-reliably: AUC=0.647 (116). CXL prognosis becomes more accurate when bioengineers combine visual acuity with other indicators.

#### 5.4.1 Clinical implication of study

The key benefit of applying AI-based techniques to this study is enhanced predictive accuracy (160). AI models, particularly ML algorithms, gain certain advantages from identifying complex patterns within large datasets (117–129). Non-linear relationships may not be apparent through traditional statistical methods. Therefore, conventional linear models can commonly miss them, but ML can capture non-linear relationships between variables (130–135). Another advantage is that ML models can continuously learn from new data, improving their accuracy and reliability over time (136–149, 161–168).

Our predictive models are accurate enough to be applied in practice. They take into account many preoperative findings to show postoperative changes in the corneal thickness. Future studies may use the same approach to build the models evidencing the efficiency of modified CXL protocols. The CXL technique has many modifications, and the analysis of the personal risk profile allows one to find the optimal intervention for the individual. Today's medicine can benefit from constructing high-performing predictive models that prognosticate treatment outcomes from preoperative findings. By improving prediction accuracy and personalizing treatment, AI can help optimize resource allocation and reduce unnecessary procedures or interventions (134, 149–153). Early and accurate predictions can lead to better patient outcomes, potentially reducing long-term healthcare costs (117, 154–156).

## 6 Conclusion

- The corneal thickness markedly drops after CXL. Transient thinning is a normal response to the treatment. The reasons behind the thinning are dehydration during surgery, keratocyte apoptosis, corneal healing, and remodeling. The postoperative changes in MCT are more pronounced, clinically relevant, and meaningful than in CCT. MCT should serve as the major clinical marker of corneal thinning after CXL.
- The postoperative changes in pachymetry tests largely depend on the disease stage. The cornea's potential to recover reduces in advanced KC. Linear and polynomial equations reveal different trends in the dynamics of corneal thickness after CXL. According to our data, the polynomial curve demonstrated the complex time-to-corneal-thickness association more reliably than the linear trendline. The findings reflect the natural course of corneal remodeling after CXL. It includes thinning immediately after CXL and stabilization with partial recovery of corneal thickness over time. MCT and CCT show progressive reduction throughout the first 20 months of observation. Afterwards, corneal thickening occurs, restoring baseline values at approximately 52 months post-surgery.
- The baseline pachymetry data can adequately reflect the intervention outcomes due to a marked association between pre- and postoperative corneal thickness, both central and minimal. Preoperative BAD deviation and topographic indices *strongly* correlate with the structural outcomes of CXL. Keratometry and refractometry data exhibit *moderate* associations with postoperative corneal thickness. The models trained on a combination of the top correlating features with clinical data and time after surgery provide the most reliable prognosis of postoperative CCT and MCT.
- BAD-Dt is a strong correlate and predictor of postoperative MCT since it indicates the thinnest point thickness of the cornea. Although used interchangeably, Dt and MCT may correlate negatively when the thinnest point is often displaced and located in the inferior-temporal region of ectatic corneas. BAD indices are the most reliable determinants of the corneal thickness after CXL. A high SHAP value of Dt serves as an explanation of the high predictive potential of this diagnostic modality.
- Preoperative topographic indices (CKI, KI, IHD, IVA) are among the top correlates of postoperative MCT; they are also an established tool for early detection and monitoring progression of KC. The predictive potential of topographic findings is slightly lower than BAD. In particular, IHD index captures vertical decentration and corneal asymmetry–the determinants of corneal thinning and remodeling after CXL. Higher IVA values indicate steeper inferior curvature, which is often associated with corneal thinning in the central regions.
- Corneal eccentricity is a keratometry finding that demonstrates a strong inverse association with CCT and MCT after CXL; it describes the rate of flattening from the center to the periphery of the cornea. The reliability of the models trained on keratometry readings is slightly lower than with the aforementioned diagnostic modalities. Keratometry findings describe surface shape but do not reflect how the cornea will respond structurally to CXL.
- Vision characteristics and refraction parameters describe the optical outcome of the disease. They show a loose correlation with pachymetry data, which explains the questionable performance of the models trained on the visiometry and refractometry findings.

## Strength and limitations

The study possesses the following strengths:

- Previous studies primarily focused on how preoperative corneal thickness influences postoperative outcomes, including visual acuity and corneal stability. Our study modeled pachymetry values from four groups of features.
- ML prediction models allow physicians to prognosticate treatment outcomes with high precision. The presented statistical models predict postoperative changes in MCT more accurately than in CCT. The clinical value of models prognosticating MCT is also higher because this parameter provides a more direct measure of the corneal region most affected by ectatic changes in keratoconus.
- The study has a strong concept consistent with the principles of precision medicine. The findings advocate for a personalized approach to candidate selection for CXL. Personal risk assessment requires a thorough inspection of patients with pachymetry, visiometry, refractometry, and topography tests. Multimodal preoperative risk assessment provides accurate estimators of treatment efficiency.

The authors recognize the following limitations of this study:

- This study was conducted at a single center and involved a population of Eastern European descent with limited racial diversity. As a result, the findings may not be broadly applicable to other geographic regions or racial groups. Additional research is necessary to evaluate the effectiveness of CXL across different races.
- The retrospective design was another weakness of the research. It might introduce biases due to potential inconsistencies in data collection over time. Nevertheless, the results align with findings from prospective studies. For instance, similar relationships between pre- and postoperative pachymetry measurements have been reported in both retrospective (157) and prospective studies (158). While prospective data collection is ideal, it is often impractical for studying large populations or long-term treatment outcomes. In such cases, retrospective studies serve as valuable tools for investigating rare conditions and evaluating the delayed effects of treatments (159).
- The current study reveals models of corneal thickness after CXL. The equations we built demonstrate the minimal and central corneal thickness dynamics after CXL. The function approximation allows us to judge immediate and delayed outcomes. Still, the postoperative dynamics in pachymetry tests largely depend on the characteristics of the study cohort. In early stages, the cornea's potential to recover its thickness after CXL is vastly higher than in advanced stages of the disease. In addition, the long-term prognosis of MCT changes may be imperfect since the maximal observation period was approximately 50 months.

## Data Availability

The raw data supporting the conclusions of this article will be made available by the authors, without undue reservation.
